# Immune Dysfunction, Cytokine Disruption, and Stromal Changes in Myelodysplastic Syndrome: A Review

**DOI:** 10.3390/cells11030580

**Published:** 2022-02-08

**Authors:** Olivia F. Lynch, Laura M. Calvi

**Affiliations:** 1 School of Medicine and Dentistry, University of Rochester, Rochester, NY 14642, USA; olivia_lynch@urmc.rochester.edu; 2 Division of Endocrinology and Metabolism, Department of Medicine, School of Medicine and Dentistry, University of Rochester, Rochester, NY 14642, USA

**Keywords:** myelodysplasia, cytokines, microenvironment, immunity

## Abstract

Myelodysplastic syndromes (MDS) are myeloid neoplasms characterized by bone marrow dysfunction and increased risk of transformation to leukemia. MDS represent complex and diverse diseases that evolve from malignant hematopoietic stem cells and involve not only the proliferation of malignant cells but also the dysfunction of normal bone marrow. Specifically, the marrow microenvironment—both hematopoietic and stromal components—is disrupted in MDS. While microenvironmental disruption has been described in human MDS and murine models of the disease, only a few current treatments target the microenvironment, including the immune system. In this review, we will examine current evidence supporting three key interdependent pillars of microenvironmental alteration in MDS—immune dysfunction, cytokine skewing, and stromal changes. Understanding the molecular changes seen in these diseases has been, and will continue to be, foundational to developing effective novel treatments that prevent disease progression and transformation to leukemia.

## 1. Introduction

Myelodysplastic syndromes (MDS) occur when mutant hematopoietic stem cells (HSCs) proliferate, giving rise to dysplastic myeloid progeny and ineffective hematopoiesis [1]. There is no one mutation responsible for the initiation of MDS; rather, a wide array of mutations involved in multiple cellular processes contribute (See Table 1 for common mutations) [2,3,4,5,6,7]. Most cases of MDS arise from at least 2–3 coexisting mutations within HSCs or progenitor cells that drive disease development [8]. MDS may arise from precursor lesions, such as clonal hematopoiesis of indeterminate potential (CHIP), where clonal populations of HSCs carry mutations also identified in hematologic malignancies [9,10]. Recently, somatic mutations have been detected in HSCs as early as in the embryo [11], including CHIP mutations [12], highlighting the importance of understanding the forces that participate in the clonal selection, including the role of microenvironmental signals.

Some of these initiating mutations are advantageous for the HSCs and allow for continued expansion, while they are detrimental to functional hematopoiesis [13]. What ensues is a deficiency of mature hematopoietic cells and an abundance of blast cells. Therefore, MDS clinically manifests with cytopenia—specifically, anemia, neutropenia, and thrombocytopenia, among others. The wide range of disease severity seen in MDS is due both to the variety of driver mutations and the extent to which clonal cells have overtaken normal bone marrow function. While some mutations such as *SF3B1* may cause a slowly progressive MDS, others, including Rat sarcoma virus (*RAS*)-pathway mutations, are associated with increased blast counts and, therefore, higher risk of progression to Acute Myeloid Leukemia (AML), which occurs when myeloblasts account for greater than 20% of nucleated cells in the bone marrow [14,15].

Notably, several common mutations do not alone provide the clone with proliferative advantage, with HSCs that are defective in their growth and differentiation in vitro and that engraft poorly in immunodeficient recipient mice [16]. For example, Isocitrate Dehydrogenase (*IDH*) mutations are epigenetic modifiers and do not have a proliferative advantage per se [17,18]. Similarly, Serine and Arginine Rich Splicing Factor 2 (*SRSF2*) mutations influence the splicing machinery and require additional proliferative mutations [19]. These findings may invoke interactions of the clone with the microenvironment, including immune cells and signals, as a mechanism of clonal expansion.

**Figure 1:**
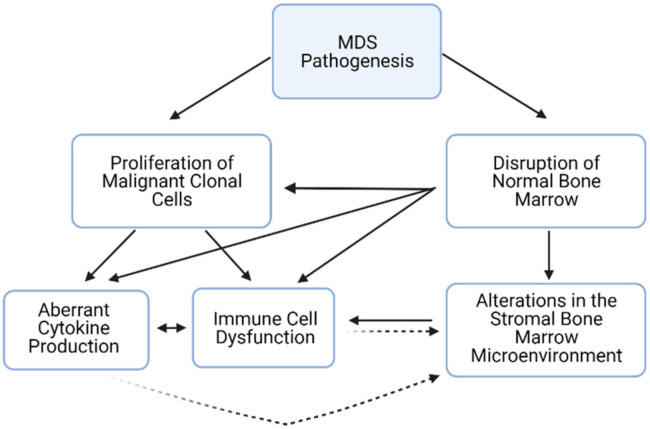
A model of the multifaceted pathogenesis of Myelodysplastic Syndromes. Solid arrows denote a direct causal relationship supported by experimental data; dashed arrows demote relationships that are hypothesized but not firmly experimentally established to date.

The manifestations of MDS are due both to the proliferation and aberrant differentiation of mutated, malignant HSCs and their progeny, as well as the failure of normal bone marrow as clonal MDS cells take over. The complex interplay between these two categories of cell populations ultimately determines the course of the disease (Figure 1). At its core, there are three facets of microenvironmental niche dysfunction that are central to MDS development and progression—immune cell dysfunction, skewed cytokine production, and stromal cell disruption.

Exploring the individual contributions of these components, as well as the significant interactions between them, will be the focus of this review. Immune dysfunction is manifested both by clonal MDS cells and previously normal mature immune cells. Cytokine production in MDS is also altered—not only by MDS cells and normal hematopoietic cells but also by stromal bone marrow cells. Finally, beyond aberrant cytokine production, stromal cells in the bone marrow microenvironment contribute significantly to disease development and progression via multiple mechanisms. The overlapping nature of these three components highlights a core principle of MDS—there is no one mutation, alteration, or microenvironment that alone initiates or facilitates disease. Rather, interactions between multiple endogenous and clonal cell populations are responsible. Given its complexity, much is still unknown regarding MDS pathogenesis; however, this review aims to synthesize key research related to immune cell behavior within the MDS microenvironment, cytokine production patterns in MDS, and stromal cell contributions to disease development and progression.

## 2. Immune Cell Dysfunction in MDS

Hematopoietic stem cells are impacted by their surroundings [20]. From chemokine and cytokine production to altered cellular processes, the inflammatory bone marrow microenvironment appears to both influence and be influenced by the presence of MDS. This section will explore both the impact of MDS on the endogenous immune system and the changes seen in clonal MDS immune cells that contribute to further inflammation. 

### 2.1. Changes in the Endogenous Immune System with MDS

Immune cells existing in the bone marrow microenvironment are altered in the presence of MDS, specifically macrophages, dendritic cells, Natural Killer (NK) cells, B cells, and T cells (Table 2). Research has shown that alterations in these cells’ behavior and proliferative abilities are associated with disease progression, with increased dysfunctional B cells and low regulatory T cells (Tregs) seen in lower-risk MDS and aberrant NK cells and increased Tregs seen in higher-risk MDS [21]. Therefore, understanding their patterns of dysfunction is critical to developing targeted therapies (Table 2). What follows is a survey of established contributions by immune cells dysfunction in MDS.

Macrophages interact with and regulate hematopoietic stem cells [22,23,24]. In addition, they provide critical support for erythropoiesis as part of the erythroblastic islands in the bone marrow [25]. Macrophages produce various cytokines and inflammatory factors, including vascular endothelial growth factor (VEGF), which stimulates angiogenesis. Research has found increases in vascular density in MDS, suggesting an indirect role for macrophages in disease development that may warrant further investigation [26]. Other studies have found a higher frequency of macrophages in MDS, which is associated with proapoptotic cytokine production [27]. Of note, apoptosis of hematopoietic precursors is a key feature of lower-risk MDS [28]. Since macrophages represent a critical phagocytic population that engulfs apoptotic cells in the bone marrow [29,30], their role may be particularly important in low-risk MDS. However, whether the phagocytic capacity of macrophages is abnormal in MDS is not known. Additionally, resident macrophages have been shown to be critical in successful HSC engraftment and growth in murine models of stem cell transplantation, suggesting their importance in normal bone marrow homeostasis and hematopoiesis [22].

Dendritic cells (DCs) represent a key component of the low-risk proinflammatory immune response. DCs are antigen-presenting cells and thus stimulators of T cells. Antigens from apoptotic HSCs in low-risk MDS are presented by DCs, therefore triggering induction of T-cell activation against normal HSCs. The ensuing apoptosis of normal HSCs contributes to the dominance of clonal MDS-derived HSCs and disease progression [21].

Natural Killer (NK) cells are responsible for the lysis of unwanted cells, and therefore have intrinsic antitumor activity. In high-risk MDS, there are decreased numbers of NK cells, allowing further clonal proliferation. In low-risk MDS, however, it appears that NK cells are cytotoxic to clonal MDS precursors, thereby curbing disease progression and presenting an opportunity for therapeutic intervention for myeloid malignancies including MDS [31,32].

B lymphocyte dysregulation is seen in autoimmune disorders; therefore, researchers have hypothesized similar findings in MDS. While some evidence has shown downregulation of B-cell signaling pathways in MDS, further research is needed to clearly delineate this relationship [33].

Multiple subtypes of T lymphocytes are altered in the setting of MDS. Firstly, regulatory T cells (Tregs) play separate and unique roles in low-risk versus high-risk MDS. In low-risk MDS, low numbers of Tregs allow T-cell activation and apoptosis. In high-risk MDS, however, it has been found that high numbers of Tregs inhibit the ability of the endogenous immune system to respond to clonal cells, thereby allowing for MDS cell proliferation [34]. Secondly, cytotoxic T lymphocytes are observed to be increased in MDS, although evidence shows that this activated response facilitates the killing of normal HSCs, thereby accelerating disease development [35].

Myeloid-derived suppressor cells (MDSCs) are becoming recognized as key cell populations derived from the normal innate immune system that, in the presence of the tumor, which often recruits these cells by secreting chemokines, provide immunosuppressive signals that suppress the adaptive immune response to cancer. This population has long been recognized in solid tumors and is beginning to be studied as part of the immune microenvironment in MDS. As in solid tumors, the data published to date show that MDSCs interfere in tumor immunity by suppressing cytotoxic T cells [36]. In addition to these immunomodulatory actions, MDSCs in MDS produce inflammatory mediators that directly disrupt erythropoiesis, contributing to disease progression [37]. Notably, Sallman et al. recently reported that patients with *TP53* mutations have an expansion in MDSCs, with an associated decrease in bone marrow infiltrating cytotoxic T cells [38]. The authors speculate that the immunosuppressive phenotype of this subset of MDS may be in part responsible for their inferior responses to therapy, highlighting MDSCs as an important potential therapeutic target, at least in specific MDS subsets. 

### 2.2. MDS Clonal Cells’ Contribution to Inflammation 

Along with alterations in endogenous immune cells, clonal MDS immune cell progeny contribute to an inflammatory environment that favors ineffective hematopoiesis. MDS-derived monocytes, macrophages, dendritic cells, and neutrophils all behave aberrantly.

Monocytes isolated from MDS patients have been shown to have a unique phenotype. Matrix metalloproteinases (MMP) are enzymes responsible for extracellular matrix remodeling and the production of cytokines. MMP-9 specifically has been found to play a role in the mobilization of HSCs and is a known secretory product of monocytic cells [39]. Research into the role of MDS-derived monocytes demonstrated reduced production of MMP-9, and further showed that decreased levels of MMP-9 were correlated with an increased proportion of clonal MDS monocytes in a dose-dependent fashion, suggesting that loss of MMP-9 may decrease HSC support by the microenvironment. Consistent with this, decreased MMP-9 levels were correlated with marrow hypercellularity—a known feature of MDS [40]. However, the role of MMP-9 is likely multifaceted. For example, MMP-9 has been shown to contribute to failed erythropoiesis in the del(5q) subtype of MDS, and likewise, MMP-9 inhibition increases colony-forming units-erythroid (CFU-E) growth [41]. While research into exact mechanisms is ongoing, this apparent suppressive activity of high MMP levels on erythroid proliferation may contribute to the link between low MMP-9 levels and hypercellularity.

MDS-derived macrophages also play a role in disease progression. Tet methylcytosine dioxygenase 2 (*TET2*) is the most frequently mutated gene seen in MDS (Table 1). Using a murine model, researchers found that *Tet2*-deficient murine macrophages had a constitutive expression of lipopolysaccharide (LPS)-induced genes, suggesting that loss of normal *Tet2* function contributes to an upregulated inflammatory state and alteration of the microenvironment [42]. Furthermore, it appears that macrophage function is itself impaired in the setting of MDS. A study looking at monocyte-derived macrophages in MDS patients found a reduction in macrophage number, impairment in phagocytosis, and a reduction of CD206 expression by macrophages [43]. CD206 is a cell surface receptor important in the engulfment of microorganisms, a critical receptor in effective phagocytic function. Despite this loss of phagocytic cell number and function, these macrophages appear to overexpress inducible nitric oxide synthetase (iNOS), an enzyme produced in response to an abundance of inflammatory cytokines, as can be observed in MDS [43]. iNOS leads to the production of nitric oxide (NO), which is a known inhibitor of bone marrow cell growth and inducer of DNA mutations; elevations of NO and iNOS are, therefore, commonly seen in other malignancies beyond MDS [43]. Via both their impaired ability to clear toxic cellular debris and their production of a molecule known to support cancer development, MDS macrophages could be contributors to disease progression. Whether these changes impact hematopoiesis and increased transformation to leukemia directly or by changing the bone marrow microenvironment in the setting of MDS remains an open question.

Dendritic cells, as previously discussed, are antigen-presenting cells that are critical to T cell activation. MDS-derived DCs have impaired function, and evidence has shown this dysfunction can dampen the immune response [44]. Further, it has been observed that DC numbers are decreased in MDS, and that a more significant decline is seen in high-risk MDS compared with low-risk [45]. Finally, research has found that MDS-derived DCs are able to activate endogenous T cells, highlighting the substantial opportunity for therapeutic intervention [46].

MDS-derived neutrophils as the product of aberrant hematopoiesis would be expected to have significant functional deficits leading to an ineffective response to infection. For example, Cao et al. demonstrated defective chemokine-dependent chemotaxis and enhanced degranulation that was associated with the expression of key structural proteins including DOCK8, Cdc42, and Rac1 in the neutrophils of 12 MDS patients [47]. This finding suggests that neutrophils in MDS are unable to migrate effectively, which may both alter the microenvironment and contribute to detrimental clinical outcomes.

## 3. Inflammation, Aberrant Cytokine Production, and MDS

The proinflammatory microenvironment in MDS that facilitates hematopoietic failure and clonal cell proliferation is, in part, secondary to the overproduction of inflammatory cytokines. Specifically, tumor necrosis factor-alpha (TNF-a), interferon-gamma (IFN-y), interleukin-6 (IL-6), interleukin 1β (IL-1B), and interleukin 8 (IL-8) are found in high levels in MDS [28]. Cytokines play a key role in facilitating cell–cell communication and interactions critical to the response to infection. Thus, altered cytokine secretion can severely disrupt cellular processes, including hematopoiesis, and contribute to the MDS phenotype. Several recent excellent reviews have provided a detailed and comprehensive presentation of the complexity of immune pathways disruption in MDS [28,48,49,50,51,52]. Here, we wish to present salient examples of key alterations in cytokine production within the inflammatory microenvironment. In the final section of the review, we explore the relevance of cytokines produced by stromal cells.

### 3.1. The Cytokine Profile of MDS

TNF-a is a proapoptotic cytokine and, therefore, likely contributes to the rapid cell death seen in lower-risk diseases [53]. As well, TNF-a-driven cytokine production has been shown to modify stromal cell gene expression, which in turn disrupts microenvironmental homeostasis [54]. IFN-y is also a proapoptotic cytokine. Additionally, IFN-y has been found to inhibit hematopoiesis and enhance iNOS production [54]. iNOS, as discussed earlier, stimulates tumorigenesis across multiple cancer types; its link to IFN-y levels further supports its role in MDS progression [43].

In MDS, alterations in transforming growth factor-beta (TGF-B) signaling, including a loss of inhibitory factors, have been shown to cause constitutive pathway activation and suppression of hematopoiesis [55]. IL-6 and IL-8 are proinflammatory cytokines elevated in many hematologic malignancies. Evidence suggests that these cytokines may serve as regulators of the microenvironment and promote sustained survival and growth of clonal cells [53,56,57].

While exact mechanisms of action are still unknown, data suggest many cytokines exert their pro-MDS effects via the nuclear factor kappa-light-chain-enhancer of activated B cells (NF-kB) pathway. NF-kB is a transcription factor that is a critical regulator of immune defense. Basal NF-kB activation is present in healthy HSCs, however, its constitutive activation induces bone marrow failure [58,59]. In MDS, increased NF-kB activation has been reported [60]. NF-kB activity, therefore, may be relevant to MDS pathogenesis [61].

These elevated cytokines also interact with immune cells, altering their behavior and contributing to many functional changes described in the prior section. For example, Tregs, which activate apoptosis in low-risk MDS and inhibit the immune response in high-risk MDS, also secrete TGF-B [34]. TGF-B, in turn, dampens hematopoiesis and allows the proliferation of clonal cells while endogenous immune cells are compromised [21]. Likewise, T-helper 17 (Th17) T lymphocytes exert their influence via the production of Interleukin 17 (IL-17), a cytokine that can, in turn, activate macrophages and DCs to produce additional proinflammatory cytokines, including IL-6 and TGF-B. Evidence has shown increased levels of IL-17 in low-risk MDS, and it is suspected to play a role in inducing apoptosis [62]. Notably, TGF-B inhibition has recently emerged as a novel approach for the treatment of MDS, highlighting the therapeutic impact of targeting the bone marrow microenvironment in MDS [63].

IL-6 is a key inflammatory cytokine that likely contributes to CHIP and MDS not only by driving clonal dynamics but also by directly mediating hematopoietic dysfunction. For example, IL6 modulates the expansion of mutated *TET2* and *DNMT3A* clones [64,65]. Moreover, in del(5q) MDS, loss of miR145 and miR146a mediates increases IL-6 through *TRAF6* overexpression as a mechanism of cytopenia and myelodysplasia [66].

MDS also appears to trigger activation of the NLRP3 inflammasome, an endogenous inflammatory system. As noted previously, apoptosis is a hallmark of lower-risk MDS. However, recent research has highlighted similarities in apoptotic gene expression between MDS patients and healthy controls, therefore suggesting that other cellular death pathways contribute to the cell lysis seen in the disease [67]. Specifically, an inflammatory cell death process termed pyroptosis has been proposed. This is based on the findings that Caspase 1 levels, which activate pyroptosis, are increased in MDS, whereas levels of Caspase 3, which participates in apoptosis, are normal [68]. Pyroptosis is facilitated via the formation of an inflammasome, which is a protein complex that recognizes cellular stressors and activates the production of proinflammatory cytokines. The NLRP3 inflammasome is active in MDS and is triggered by the production of reactive oxygen species generated by the danger-associated molecular protein (DAMP) S100A9, among others [50,69]. As further evidence that the NLRP3 inflammasome is activated in MDS, neutralization of S100A9, inhibition of NLRP3, and elimination of Caspase-1 all have been shown to suppress pyroptosis and improve the hematopoietic failure seen in MDS [68]. In addition to these direct effects, NLRP3 activation also activates IL-1B, a cytokine produced by defective macrophages in CHIP [70,71] and aging [30], and a known mediator of HSC skewing [72]. Notably, NLRP3 and IL-1B are therapeutically targetable and may therefore represent a novel strategy for MDS treatment.

### 3.2. Prognostic and Therapeutic Significance of Cytokine Imbalance

Increasing evidence suggests that cytokine levels can be predictive of clinical outcomes in certain circumstances. A study of plasma cytokine levels in MDS patients found that increased levels of IL-6, chemokine (C-X-C motif) ligand 10 (CXCL10), and Interleukin 7 (IL-7) predict shorter overall survival independent of cytopenias. IL-6 and CXCL10 were also predictive of worse leukemia-free survival, suggesting that elevated levels of these cytokines may contribute to leukemic progression [73]. Elevated levels of chemokine (C-X-C motif) receptor type 2 (CXCR2)—the IL-8 receptor—were also found in MDS stem cells and correlate with worse prognosis [74].

Despite this evidence, utilizing cytokine profiles as prognostic tools is challenging due to the multiple mutations and etiologies that may drive the overproduction of a given cytokine in MDS [28]. Indeed, a study of serum cytokines in MDS and AML patients found that while levels differed substantially between controls and diseased samples, no single cytokine was significantly predictive of overall survival [75,76]. However, certain cytokine signatures—groupings of samples expressing similar proportions of certain cytokines— were associated with prognostic outcomes. Nine signatures were identified, each with varying levels of eleven key cytokines—C-C Motif Ligand 3 (CCL3), C-C Motif Ligand 5 (CCL5), Platelet-Derived Growth Factor -BB (PDGF-BB), Interleukin-4 (IL-4), Interleukin-15 (IL-15), Colony Stimulating Factor 2 (CSF2), TNF-a, Fibroblast Growth Factor 2 (FGF-2), IL-17, and Interleukin 12 (IL-12). Of the nine signatures identified, eight were associated with distinct prognostic outcomes and could be grouped into favorable, intermediate, and unfavorable. A favorable cytokine signature—those with higher levels of IL-4 and CCL3—was significantly associated with higher disease remission rates [75,76].

Given the important role cytokine skewing plays in altering the inflammatory microenvironment, cytokines represent an opportunity for therapeutic intervention. However, low- and high-risk MDS have different cytokine profiles, and therefore, require distinct treatment approaches. For example, therapies targeting proapoptotic cytokines may be beneficial in low-risk MDS but not in high-risk diseases. As TNF-a is a key proapoptotic factor, monoclonal antibodies targeting TNF-a such as Infliximab and Etanercept may theoretically be beneficial; however, studies testing their use in low-risk MDS have found variable to minimal impact [77,78].

Beyond monoclonal antibodies, lenalidomide has been found to reduce TNF-a, IL-6, and IL-1B levels and is approved for the treatment of the del(5q) subset of MDS—one of the most common cytogenetic alterations seen in MDS [79]. It also induces apoptosis in clonal MDS progenitor cells and modulates endogenous immune cell behavior. As well, the effects of erythropoietin (EPO)—given to MDS patients to combat anemia and defer blood transfusions—are suppressed by TNF-a, and it has been found that giving lenalidomide with EPO improves this unwanted suppressive effect [80,81]. Given its multifactorial potential benefit, including its ability to modulate skewed cytokine production, lenalidomide is the treatment of choice for MDS patients with 5q deletion.

While it is clear that certain cytokines are upregulated or downregulated in MDS, and therapeutic modulation of cytokine levels can be moderately effective in managing disease, there is still much that is unknown about why and how certain cytokines impact disease progression. Namely, the impact of common MDS mutations (Table 1) on cytokine production and the link between certain cytokine profiles and clinical and prognostic outcomes is still an active area of research. 

## 4. The Stromal Microenvironment

The ability of the bone marrow microenvironment to regulate hematopoietic stem and progenitor cells is well-established. While not the focus of this review, more information on these interactions can be found in recent review articles, including those by Ho et al., 2020, Pinho et al., 2019, and Galán-Díez et al., 2018 [82,83,84].

### 4.1. Stromal Contributions to the Propagation and Initiation of Hematopoietic Failure

MDS is no longer considered a disorder of only hematopoietic cells but rather a dysfunction of the entire bone marrow, including mesenchymal components. Early research looking at bone cell function in MDS patients found close relationships between hematopoietic and stromal cells [85]. Mineral apposition rate—a dynamic measure of bone formation—was significantly decreased in MDS patients compared with controls, as was total osteoclast and osteoblast number. Combined with data showing normal osteoid but decreased osteoblast surface area, these findings suggested a deficiency in osteoblast development, recruitment, or efficiency in the setting of MDS, suggesting that the function of mesenchymal cells is linked to that of hematopoietic populations [85].

In fact, functioning osteohematopoietic interactions and stromal support are necessary for maintaining healthy hematopoiesis. Mesenchymal stromal cells (MSCs) are critical to hematopoietic differentiation. This link has been further demonstrated by the fact that MDS-derived MSCs are unable to provide stromal support, contributing significantly to hematopoietic deficits seen in MDS. Human bone marrow fibroblast colony-forming units (CFU-F) assays performed demonstrated reduced CFU-F counts in MSCs from MDS patients compared with those purified from healthy controls. MSCs from these MDS samples also had impaired growth in culture and were not able to be maintained at as high passages as samples from healthy controls. MDS-derived MSCs also were more likely to senesce. As evidence of the molecular differences underlying these observed phenotypic changes, differential methylation was observed between the MDS-derived and healthy bone marrow MSCs. Specifically, hypermethylation was seen in promoter regions of DNA within the MDS MSC samples, resulting in the silencing of genes associated with key cellular processes. These deficits translate into decreased support of stem cell populations; in a long-term coculture with CD34+ hematopoietic stem and progenitor cells (HSPCs), MDS-derived MSCs compared with MSC controls had substantially reduced ability to provide HSPC support. This deficiency was able to be restored when the CD34+ HSPCs were then cocultured with healthy MSCs. The molecular mechanisms of this defective support include differential cytokine expression by MDS-derived MSCs and shifting of HSPCs into the resting phase of the cell cycle by MDS MSCs [86].

Not only is MSC support needed for functional hematopoiesis, but further research by Raajmakers et al. utilizing a murine model of *Dicer1* deletion demonstrated that a dysfunctional stromal environment could initiate myelodysplasia [87]. *Dicer1* deletion downregulates the production of miRNA, which is essential to hematopoietic differentiation and maturation. By introducing this gene deletion into mesenchymal osteolineage cells of mice via Cre recombinase, the effects of *Dicer1* gene deletion on osteoprogenitor cell growth and differentiation could be studied. The deletion was seen in MSCs but not in HSPCs, and classic features of human MDS were observed. The osteogenic population was adversely impacted, similar to findings in previously mentioned studies, with decreased CFU-F counts and reduced levels of differentiation markers such as osteocalcin. However, this study uniquely demonstrated the causal link between altered MSCs and hematopoiesis, with mutant mice experiencing profound anemia and thrombocytopenia with altered hematopoietic cell morphology. Moreover, the bone marrow cellularity of these mice was normal to hypercellular, and HSPC populations were not adversely impacted in terms of count or function. These mice also had reduced B cells and increased myeloid cells. All of these findings are characteristic of human MDS. Several of these mice subsequently developed AML—another key feature of progressive MDS. Researchers then transplanted HSPCs from these mutant mice into wild-type mice and were able to demonstrate normal cell counts and a lack of myelodysplasia, showing the inability of HSPCs alone to trigger an MDS phenotype. However, coculturing MSCs from mutant mice with normal HSPCs in vitro demonstrated morphologic and functional changes in the hematopoietic cells, supporting the idea that altered osteogenic cells directly alter HSPC function.

The ability of MSCs to drive hematopoietic failure in humans, however, has not yet been established and is a challenge to study. Xenotransplantation of human MDS into mouse models is a method that hypothetically would allow for longitudinal, controlled examination of the interactions between the stroma and hematopoietic cells throughout the disease course. Yet, the engraftment of human MDS cells to murine models has proved difficult by several studies, perhaps suggesting the importance of the microenvironment in the propagation of the disease. Engraftment has shown to be improved, however, with the addition of stromal cells [88,89]. Furthermore, the specific stromal cells found to facilitate engraftment—Human skin fibroblast cell 27a (HS27a) cells—express the CD146 cell surface marker known to be critical in MDS clonal expansion [88].

The concept of donor cell leukemia (DCL) provides further support for the importance of the stromal environment in initiating hematopoietic failure. DCL occurs when a patient transplanted with supposedly normal donor cells develops hematologic malignancy, but the donor does not. Researchers have speculated that dysregulation of the bone marrow microenvironmental niche alters the differentiation and proliferation pathways of HSPCs, possibly to such an extent that they become leukemogenic [90]. Cytokine and cellular signaling pathways are known to be disrupted by the chemotherapy and radiation that occurs preceding the bone marrow transplantation and, therefore, may trigger the alteration of the microenvironment [91]. Extrapolating the phenomenon of DCL to hematopoietic failure generally provides support for the stromal environment as an important component of MDS initiation. However, undetected CHIP in donors provides an alternate explanation for the development of DCL in these patients [92,93,94]. On the other hand, even in this case, the malignant stromal may indeed facilitate the emergence of the CHIP clone.

Recent research provides further rationale for targeting the bone marrow microenvironment as a therapeutic strategy. Using a murine model of MDS, Balderman et al. found that normal cells exposed to the same microenvironment present in MDS acquire myeloid skewing [95]. Rejuvenation of the microenvironment via transplantation of healthy bone marrow mitigated some of the bone marrow failure and leukemic transformation seen in MDS [95]. These data provide a strong rationale for targeting the bone microenvironment to delay and mitigate MDS progression.

The potential of the bone marrow microenvironment as a therapeutic target in MDS was recently demonstrated by the approval of Luspatercept, an activin receptor type IIB, for the treatment of low-risk MDS. Wobus et al. found that bone marrow-derived stromal cells from patients treated with Luspatercept, who had improved erythropoiesis, were superior in their ability to support normal hematopoiesis, with normalization of CXCL12 production in the MDS microenvironment [96]. This study highlights the importance of assessing the microenvironment as an outcome and target of treatments in MDS.

### 4.2. Mechanisms of MSC–HSPC Interaction

The exact mechanisms underlying how stromal cells interact with and influence hematopoietic cells to contribute to their failure in MDS is an active area of research. Activation of Wingless-related integration site (Wnt) signaling in MSCs has been shown to drive bone marrow failure. Secondly, as previously discussed, shifts in cytokine production by both non-MDS and clonal MDS immune cells contribute to the MDS phenotype; however, research has shown that cytokine skewing by mesenchymal cells, including cytokine production initiated by the NF-kB pathway, is also relevant. Ultimately, it is likely the interplay between these various mechanisms that is integral to disease development. 

The Wnt/B-catenin signaling pathway is critical in determining cellular fate, migration, and proliferation. This was initially observed in embryonic development and the importance of the pathway in establishing the body axis [97]. More recently, it has been found to play a critical role in carcinogenesis, especially in myeloid neoplasms. Bhagat et al. found that an antagonist of the pathway—the Frizzled Related Protein (*FRZB)* gene—was hypermethylated, and therefore its expression was suppressed in the stromal cells of MDS patients, allowing increased activation of the Wnt pathway. Furthermore, primary MDS-derived MSCs had increased activation of this pathway. The Nup98-HoxD13 (*NHD13*) fusion gene has been identified in subsets of patients with MDS; when mice transgenically express this gene under the control of the Vav promoter, they develop prolonged cytopenias, eventually progressing to leukemia [95,98]. While *NHD13* mice are frequently used to model MDS, its phenotype is more representative of low-proliferative Acute Myeloid Leukemia (AML) than MDS. In this experiment, *NHD13* mice were crossed with constitutively-active human B-catenin mice models (*S33Y*), and the bone marrow of these mice was transplanted into irradiated recipients who were followed for disease progression. Most *NHD13/S33Y* mice died from myeloid leukemia, whereas other nonmutant mice died of a less aggressive MDS-like phenotype, suggesting the importance of Wnt/B-catenin activation in leukemic transformation. To apply these findings to human MDS samples, researchers determined the frequency of transcription of genes downstream of the Wnt pathway in healthy controls and CD34+ cells from the bone marrow of MDS patients. Many of these genes were found to be overexpressed in the MDS patients compared to controls. Furthermore, higher-risk MDS cases (as determined by blast counts) had higher levels of transcription of gene products downstream of the Wnt pathway, suggesting an almost dose–response effect of pathway activation. These findings were also extremely clinically relevant—patients who had a high level of Wnt pathway activation had a median overall survival of 2.95 years compared with a median survival of 5.24 years in those with a low Wnt activation profile. Taken together, these data highlight the importance of Wnt/B-catenin activation in disease progression and leukemic transformation [99].

Further evidence of the importance of this pathway comes from Stoddart et al., who found that inhibition of the Wnt/B-catenin pathway can prevent MDS development. Adenomatous polyposis coli (*Apc*) is a negative regulator of the Wnt signaling pathway, and prior research by this group demonstrated that mice deficient in *Apc* developed MDS. Researchers were then able to show that loss of a single copy of the *Cttnb1* gene (which encodes B-catenin, a Wnt pathway transducer) was able to prevent MDS in this same *Apc*-deficient mouse model, suggesting that this pathway is not only able to induce MDS when upregulated, but that suppression of the pathway can inhibit MDS development. For further validation of these findings, pyrvinium—an FDA-approved medication that inhibits the Wnt pathway—was given to these *Apc*-deficient mice, and MDS development was likewise prevented [100].

Altered cytokine expression is another mechanism by which mesenchymal cells influence the development of MDS. Specifically, activation of the NF-kB pathway, and therefore, overexpression of inflammatory cytokines, adversely impacts hematopoiesis. Ping et al. found significantly more enriched inflammatory genes in MDS mesenchymal cells compared with normal MSCs. Furthermore, observation of the NF-kB inhibitor NFKBIA—autoregulated by NF-kB and therefore reflecting activation levels of the signaling pathway—demonstrates overexpression in the majority of MDS patients’ MSCs. Activation of the NF-kB pathway in these mesenchymal cells was also significantly associated with increased production of downstream cytokines, including IL-6, IL-8, CCL3, CCL5, S100 calcium-binding protein A9 (S100A9), and Inhibin subunit beta A (INHBA), which are known suppressors of hematopoiesis [101].

Initiation of inflammatory programs via other activation mechanisms appears to contribute to the MDS phenotype as well. CD271+ cells are mesenchymal bone marrow cells that exist in close proximity to CD34+ HSPCs. Transcriptional analysis was performed on sorted MDS stromal cells and found enhancement of inflammatory signatures in CD271+ cells. Overexpression of inflammatory cytokines, and those known to be negative regulators of hematopoiesis, including IL-6 and IL-8, were found in MDS stromal cells compared with normal controls. This data both supports the idea that stromal cell cytokine production has the potential to cause the hematopoietic failure seen in MDS and that spatial location of cells relative to one another may matter, as the CD271+ cells are in close proximity to HSPCs [102].

Finally, MSCs may also modulate immune populations in MDS. For example, Serhan et al. found that MSCs from patients with MDS could alter monocytes, inducing phenotypic and metabolic changes consistent with MDSCs, which could then suppress NK cells. [103]. These data suggest that MDS-associated MSCs may orchestrate immunosuppression through their interaction with monocytes.

As Pronk and Raaijmakers, 2019 highlight, these mechanisms underlying mesenchymal–hematopoietic interactions overlap and influence one another, creating a microenvironment prime for bone marrow failure. For example, Wnt signaling is upregulated by inflammatory cytokines and activated by NF-kB. Moreover, NF-kB impairs the proliferation of MSCs and promotes cellular senescence. Cell senescence induces the production of inflammatory cytokines, which, in turn, upregulates Wnt signaling pathways. Therefore, these mechanisms not only directly mediate mesenchymal cells’ ability to support hematopoiesis but also influence other inflammatory processes, creating spirals that result in an exponentially more toxic microenvironment [104].

As this review has highlighted, the impact of various components of the bone marrow microenvironment on MDS development and progression is an exciting and active area of research with clinical and therapeutic implications. Several critical areas of research are important to explore, especially relating to the role of key MDS mutations (Table 1). For example, understanding how the mutational burden impacts the microenvironmental response and what the mechanisms are that drive these mutations to alter cellular function and contribute to marrow failure has the potential to change the landscape of treatment options for this disease.

## 5. Conclusion

Alterations in the three core components discussed in this review—immune cells, inflammatory signals and cytokine production, and the stromal microenvironment—contribute to the range of disease phenotypes and clinical manifestations seen with myelodysplastic syndromes. The mutational and clinical heterogeneity in MDS presents a challenge to developing a single sweeping and successful therapeutic intervention. Therefore, understanding precisely the inflammatory and microenvironmental changes seen at various disease stages with various driving mutations is necessary to construct targeted treatments to combat the proinflammatory environment of the disease and ultimately halt disease progression.

## Figures and Tables

**Table 1:** Common mutations in Myelodysplastic Syndromes (at least ≥9% frequency) and their prognostic relevance.

Mutated Gene	Mechanism of Action	Prognostic Significance
*TET2*	DNA methylation, histone modification	Unclear significance
*SF3B1*	RNA splicing	Improved survival
*SRSF2*	RNA spicing	Worsened survival
*DNMT3A*	DNA methylation, histone modification	Worsened survival
*U2AF1*/ *U2AF35*	RNA spicing	Worsened survival
*TP53*	DNA repair	Worsened survival
*RUNX1*	Transcriptional regulation	Worsened survival
*ZRSR2*	RNA splicing	Unclear significance
*IDH2*	DNA methylation, histone modification	Worsened survival

**Table 2:** Current and proposed therapeutic targets in Myelodysplastic Syndromes.

Therapy	Impact on MDS Development and Progression
NLRP3 Inhibitor	Improves hematopoietic failure, suppresses pyroptosis
Caspase-1 Inhibitor	Improves hematopoietic failure, suppresses pyroptosis
TGF-B Inhibitor	Dampens proinflammatory immune response
TNF-a Inhibitor	Suppresses apoptosis seen in lower-risk MDS
Lenalidomide	Reduces TNF-a, IL-6, and IL-1B levels in del5q MDS
Erythropoietin	Combats anemia seen in MDS
Pyrvinium (Wnt pathway inhibition)	Prevents MDS development
